# Mental Illness Stigma among professionals at a Portuguese Medical Center

**DOI:** 10.1192/j.eurpsy.2023.769

**Published:** 2023-07-19

**Authors:** D. F. Rodrigues, C. Adão, A. S. Sequeira

**Affiliations:** Psychiatry, Centro Hospitalar de Lisboa Ocidental, Lisboa, Portugal

## Abstract

**Introduction:**

Mental Illness Stigma is a barrier in access to healthcare. Stigma also influences population health outcomes by worsening, undermining adequate processes. The healthcare professionals show several stigmatising behavirous and cognitions, which may impair the adequate provision of care of this population with mental illness.

**Objectives:**

We aimed to measure mental health stigma in healthcare professionals at a portuguese hospital center.

**Methods:**

A cross-sectional study of health profissionals was performed using a survey that included socio-economic and job related questions, personal and familiar questions regarding mental health, and Attribution Questionnaire 27 (AQ-27), a translated and validated stigma questionnaire with nine stigma sub-scales (Responsability, Pity, Anger, Dangerousness, Fear, Help, Coercion, Segregation and Avoidance).

**Results:**

The sample included a total of 388 participants. The majority of the respondants were female (82,5%). The age ranged from 22 to 69 (mean = 40,05). According to the job place distribution, we found statistically significant differences in various stigma subscales among several healthcare settings within our center. The inpatient unit professionals showed lesser stigmatising attitudes in anger, coercion, segregation and avoidance domains; and higher stigmatising attitudes in pity and help domains. However, professionals who work at surgery room showed higher stigmatising attitudes in danger and fear, but lesser levels of help domains. We also found differences in five stigma subscales among various health professions. The study didn’t show differences in stigma domains regarding personal or professional contact with mental illness, neither academic studies in mental health.

**Conclusions:**

Our findings suggest that workplace environment and profession may impact mental ilness stigma levels in healthcare professionals. We propose that future studies could be done to investigate methods to mitigate mental illness stigma, tailored to address different stigma domains in different workplace settings.

**Disclosure of Interest:**

None Declared

